# FAHFAs are detected in postprandial chylomicron and VLDL fractions and can be released from TG estolides by LPL in vitro

**DOI:** 10.1016/j.jlr.2026.101059

**Published:** 2026-05-20

**Authors:** Dovile Milonaityte, Kristyna Brejchova, Jaroslav Srp, Eva Kudova, Hana Chodounska, Laurence Balas, Thierry Durand, Nada A. Abumrad, Ondrej Kuda

**Affiliations:** 1 Institute of Physiology, Czech Academy of Sciences, Prague, Czech Republic; 2 Institute of Organic Chemistry and Biochemistry, Czech Academy of Sciences, Prague, Czech Republic; 3 Institut des Biomolécules Max Mousseron (IBMM) UMR 5247, CNRS, UM, ENSCM Pôle Chimie Balard Recherche Montpellier, Montpellier, France; 4 Department of Medicine, Division of Nutritional Sciences and Obesity Research, Washington University School of Medicine, St Louis, MO, USA

**Keywords:** Fatty acid/Transport, VLDL, Lipoproteins, Lipidomics, Lipase

## Abstract

Fatty acid esters of hydroxy fatty acids (FAHFAs) are bioactive lipids with antidiabetic and anti-inflammatory effects, but the mechanisms responsible for their transport in blood are not well understood. In this study, we investigated potential serum carriers of FAHFAs. Microscale thermophoresis showed that binding of 9-PAHSA to serum albumin did not support albumin as the dominant carrier under the conditions tested. We therefore turned our attention to lipoproteins and extracellular vesicles, which were isolated from murine serum by density-based ultracentrifugation and characterized by proteomics and lipidomics. Lipidomic analysis revealed that chylomicrons, very-low-density lipoproteins (VLDL), and low-density lipoproteins (LDL) contain both FAHFAs and their storage form, triacylglycerol estolides (TG-EST). In contrast, no FAHFA-containing lipids were detected in serum extracellular vesicles. As a validation case study, we confirmed the presence of TG-EST in postprandial chylomicrons from human volunteers. Functional assays further showed that lipoprotein lipase (LPL), the enzyme that hydrolyzes triacylglycerols during lipoprotein metabolism, releases FAHFAs from TG-EST. LPL exhibited positional selectivity, releasing distinct PAHSA regioisomers with different efficiencies. Together, these results support the interpretation that FAHFA-related lipids are associated with chylomicron, VLDL, and LDL fractions in the postprandial setting and establish LPL-mediated lipolysis as a potential mechanism for the release and distribution of these bioactive lipids in the circulation.

## Graphical abstract

**Figure:**
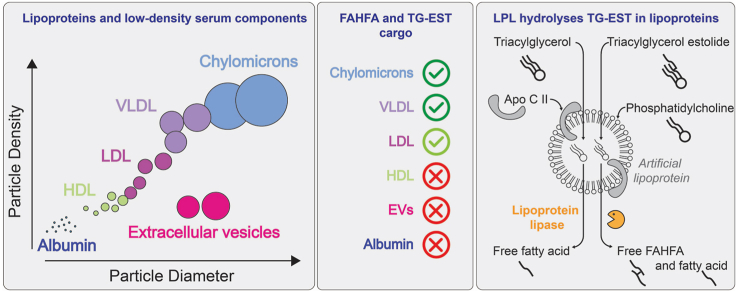

Branched fatty acid esters of hydroxy fatty acids (FAHFAs) are a class of bioactive lipids with beneficial health effects (1). They consist of a fatty acid (FA) attached to a hydroxy FA through a branched ester linkage to the hydroxyl group. Thus, FAHFAs are structurally related to oxidized fatty acid derivatives such as eicosanoids, as both arise from hydroxylated fatty acid intermediates (2). The FAHFAs have a diverse molecular composition (1, 3) and are obtained from the diet (4, 5, 6, 7, 8, 9, 10, 11) or synthesized in the body by adipose triglyceride lipase (ATGL) in liver and adipose tissue (1, 12, 13, 14, 15). Some members of the FAHFA family were shown to have strong anti-diabetic effects. For example, palmitic acid esters of hydroxystearic acid (PAHSAs) were found to correlate positively with insulin sensitivity (1, 12, 16). FAHFAs also have been documented to have anti-inflammatory effects in adipose tissue (1, 17), immune cells (18), hepatocytes (19), and in intestinal colitis (20).

Most detected FAHFAs in the body are stored esterified to a glycerol backbone as a component of triacylglycerol estolides (TG-EST) (21, 22). ATGL is responsible for the production and remodeling of TG-EST, and for FAHFA release in adipocytes (15, 23). However, how FAHFAs are transported in the body to be stored, or act is unknown.

Transport of common FAs in the blood occurs in two major forms: free or esterified. Non-esterified free FAs released by adipocytes in response to energy demand circulate bound to hydrophobic pockets in serum albumin (24, 25). Although total serum FA concentrations are high in the mM, the unbound FA in equilibrium with albumin is at nanomolar concentration, for example, 7.5 nM (26).

Esterified FAs circulate as components of the TG of chylomicrons produced by intestinal enterocytes and the TG of very low-density lipoproteins (VLDL) produced by the liver. To form chylomicrons, enterocytes reincorporate the absorbed FAs into TGs with the formation of TG-rich lipid droplets in the endoplasmic reticulum (27, 28). Mature chylomicrons, containing apolipoprotein B-48, exit the Golgi in large transport vesicles, are exocytosed across the enterocyte basolateral membrane into the lamina propria, and from there enter intestinal lymphatic lacteals (29, 30). Lymph chylomicrons reach the blood at the left subclavian vein and deliver TGs to target organs (28, 31).

Similarly, VLDLs are produced from endogenously synthesized FAs in the liver. The FAs are incorporated into TG and form primordial VLDL in the rough ER by lipidation of Apo B-100, a variant of Apo B-48. Primordial VLDL fuse with the TG-rich droplets, undergo further processing in the Golgi, and mature VLDL are released directly into the blood (32).

To deliver the FAs to tissues, the TG-rich chylomicrons and VLDL are hydrolyzed by lipoprotein lipase (LPL). LPL is secreted by adipocytes, myocytes, and macrophages (33), and its secretion and function are highly regulated (34). The major LPL producers are adipocytes and adipose LPL transfers to the luminal face of capillary endothelial cells, where its activation by Apo C-II on lipoproteins (35, 36) initiates hydrolysis of circulating TG. The liberated FAs are taken up by tissues and used for mitochondrial oxidation or stored for later use (35). As chylomicrons and VLDL lose most of their TG, remnant particles are cleared by the liver, although some VLDL remnants are processed into intermediate-density lipoproteins and subsequently into the low-density lipoproteins (LDL), enriched in cholesteryl esters (CE) (37). Unlike chylomicron and VLDL, high-density lipoproteins (HDL) are poor in TG and primarily transport CE. HDL are not hydrolyzed by LPL (38).

We hypothesized that FAHFAs are transported in the bloodstream by carriers similar to those used for simple fatty acids. Specifically, they are either bound to serum albumin or incorporated as compound lipids into (lipoprotein) particles.

## Materials and methods

### Animals, diet, and serum collection

Male C57BL/6 mice were maintained at room temperature with a 12-h light/dark cycle and free access to water and food. The mice were fed a standard diet (67% metabolizable energy from carbohydrates; ssniff; V1536). Mice were sacrificed 8 h after fasting, and blood was collected immediately to prevent metabolic changes. Samples were used to optimize lipoprotein and extracellular vesicle preparations. High-fat diet (HFD) experiments involved feeding mice starting at 13 weeks of age a diet with 60% metabolizable energy from fat (ssniff; E15742-34). Before blood collection, mice were switched to a standard diet for three days, then fasted overnight. The following day, mice were re-fed the HFD for 1 h, followed by gavage with 400 μl of 40% cream. This feeding treatment was chosen to maximize the lipid content in mouse blood coming from dietary sources, as both HFD oils and dairy cream contain FAHFAs (6, 39). Blood was collected 1 h later via the portal vein from anesthetized mice, left to clot for 30 min, centrifuged at 2,000 x *g* for 10 min, and serum was collected. The portal vein was used to maximize blood yield per mouse.

All animals are housed in facilities accredited for the use of laboratory animals by the Ministry of Agriculture of the Czech Republic. Experiments are conducted under veterinary supervision with full compliance with Act No. 246/1992 Coll., on Protection of Animals Against Cruelty, as amended, and Decree No. 419/2012 Coll., on Protection of Experimental Animals, as amended. These enactments implement Directive 2010/63/EU of the European Parliament and of the Council on the protection of animals used for scientific purposes.

### Human study

Three healthy volunteers underwent a 12 h overnight fast. The following morning, serum TG content was increased via an oral fat load in the form of cream (33% fat content). Each volunteer consumed enough cream to receive 50 g of fat per m^2^ of body surface area, which takes into the account the weight and the height of the volunteers, and has been previously shown to cause blood TG peak at the 2 h mark (40). Two hours after the oral fat load, 9 ml of venous blood was collected into silica-coated blood collection tubes (Greiner Bio-One Vacuette; 22040009). Serum was prepared by leaving the blood to clot at room temperature for 30 min, followed by centrifugation at 2,000 x *g* for 10 min at 4 °C. Serum was split into 600 μl aliquots and stored at −80 °C until further processing. Lipoprotein fractions were isolated using the same ultracentrifugation workflow used for the mouse samples. Lipidomics analysis was performed using the same analytical platform as for the mouse fractions. Experiment was approved by the Ethics Committee of the Institute for Clinical and Experimental Medicine, Prague, #61084-A-25-32 and the Ethics committee of the Institute of Physiology, CAS, Prague, #12/2025 EC-IPHYS “Isolation of Lipoprotein Particles from Human Serum”. Experiment was in agreement with the Declaration of Helsinki principles. All participants provided written informed consent.

### Microscale thermophoresis (MST)

The binding affinity of bovine serum albumin (BSA, FA-free; Sigma-Aldrich, A8806-5G) to palmitic acid (PA; Sigma-Aldrich, P0500-10G) or 9-PAHSA (Cayman Chemicals, 17037) was measured using the Monolith NT.LabelFree (NanoTemper Technologies, GmbH). FAs were first dissolved in ethanol. BSA concentration was kept at 1 μM, and a 16-step dilution series was prepared with varying concentrations of FAs up to 24.4 μM for both PA and 9-PAHSA. The sample buffer was PBS (pH 7.4) and 10% ethanol. High ethanol content did not affect BSA intrinsic fluorescence. BSA and binding lipids were mixed and incubated overnight at 4 °C. The coupling procedure was adapted for the lower solubility of FAHFA in water compared to PA. Measurements were performed at 25 °C. The data were analyzed and plotted using the GraphPad Prism 10 software. All measurements were performed in technical triplicate.

### Lipoprotein particle isolation

In a thick-wall polycarbonate ultracentrifuge tube (Beckman Coulter, 343,778), 600 μl of mouse serum was mixed with 300 μl of 1.006 g/ml density solution (0.195 M NaCl, 0.001% EDTA-2Na, 1N NaOH). The samples were spun in a Beckman Optima MAX 130K ultracentrifuge (Beckman Coulter) equipped with an MLA-130 rotor at 664,000 × *g* (*r*_max_) for 89 min (Acceleration 1, Deceleration 3) at 16 °C. From the very top, 300 μl chylomicron/VLDL fraction was removed. The bottom fraction was mixed with 300 μl of 1.182 g/ml density solution (0.195 M NaCl, 0.001% EDTA-2Na, 1N NaOH, 2.44 M NaBr), and ultracentrifuged at 664,000 × *g* (*r*_max_) for 142 min (Acceleration 1, Deceleration 3) at 16 °C. The top 300 μl fraction containing LDL was removed. The remaining sample was mixed with 300 μl of 1.478 g/ml density solution (0.195 M NaCl, 0.001% EDTA-2Na, 1N NaOH, 7.65 M NaBr). The sample was ultracentrifuged at 664,000 × *g* (*r*_max_) for 248 min (Acceleration 1, Deceleration 3) at 16 °C. The top 300 μl fraction containing HDL was removed, while the bottom 600 μl fraction was retained as an albumin fraction. For VLDL/chylomicron separation, the 300 μl fraction containing the particles was mixed with 300 μl of 1.006 g/ml density solution, and overlayed with 300 μl of MilliQ water. The sample was ultracentrifuged at 664,000 × *g* (*r*_max_) for 142 min (Acceleration 1, Deceleration 3) at 16 °C. The top 300 μl fraction containing chylomicrons was collected, while the bottom 600 μl fraction was retained as a VLDL fraction.

### Proteomics

Isolated lipoprotein particles were processed according to the SP4 no glass bead protocol (41). Briefly, samples were solubilised with SDS [final concentration 1.5% (w/v)] in 100 mM TEAB (triethylammonium bicarbonate), reduced with 10 mM TCEP [tris(2-carboxyethyl)phosphine], alkylated with 40 mM CAA (chloroacetamide), and digested with Lys-C + trypsin. Resulting peptides were desalted on C18 StageTips, dried in Speedvac, and dissolved in 0.1% TFA + 2% acetonitrile. About 500 ng of peptide digests were separated on a C18 column using nanoUHPLC (Dionex Ultimate 3000) and analysed in a DIA mode on an Orbitrap Exploris 480 mass spectrometer equipped with a FAIMS unit. Thermo raw files were processed and visualised in Spectronaut 19.9 (Biognosys) software using the default settings with Precursor and Protein Q-value and PEP cutoff set at 0.01. Mouse reference proteome UP000000589_10090.fasta (UniProt release 2025_01) was used.

### Lipoprotein lipase hydrolysis assay

TG-EST substrates containing 5-, 7-, and 9-PAHSA regioisomers, esterified at the *sn*-1/-3 or *sn*-2 position of the glycerol backbone, were synthesized as previously described (23). Assay buffer (200 mM Tris, 130 mM NaCl, 3.3 mM CaCl_2_, pH 8.2) containing 500 μM of TG-EST or TG (glyceryl trilinoleate; Sigma-Aldrich, T9517-50MG, TG 18:1(9Z)/16:0/18:1(9Z), Sigma-Aldrich, D1657-25MG) substrate and 70 μM of phosphatidylcholine (PC; Sigma-Aldrich, P3556) was emulsified by sonication. To the substrate, Apo C-II (Sigma-Aldrich, A7910) solution was added, and the vials were incubated at 25 °C for 1 h with shaking. The TG-EST/TG Apo C-II solution was mixed 1:1 with reaction assay buffer containing 10% FA-free BSA (MP Biomedicals, 152401) and 1 μg/ml of LPL (Sigma-Aldrich, L2254-1KU). The final reaction mixture contained 250 μM TG-EST or TG substrate, 35 μM PC, 5% FA-free BSA, 0.14 μM Apo C-II, 500 ng/ml of LPL or no LPL for control samples. A parallel experiment with heat-inactivated LPL was performed with a TG. The samples were incubated at 37 °C for 60 min with shaking and put on ice before extraction.

### Sample extraction for lipidomics

The samples for lipidomics were extracted using a biphasic solvent system of cold methanol, methyl *tert*-butyl ether, and 10% methanol in water (v/v) as previously described (42), adapted for enzymatic assays (43). The upper organic phase aliquots were collected, evaporated, and resuspended in methanol with the internal standard [12-[(cyclohexylamino) carbonyl]amino]-dodecanoic acid], followed by analysis using lipidomics platforms in positive and negative ion modes.

### Solid phase extraction (SPE) of FAHFAs

Purified lipoprotein fractions were mixed with 3 ml of water and 3 ml of dichloromethane (DCM), [^13^C_4_]-9-PAHSA internal standard was also added. The glass vials were centrifuged at 3 000 × *g* for 10 min, and the bottom DCM fraction was collected and dried. SPE columns (500 mg silica, 6 ml, Thermo Fisher Scientific) were conditioned with 10 ml hexane. Extracted lipids were resuspended in 300 μl of chloroform and loaded into the column. Interfering TGs were eluted with 10 ml of 5% ethyl acetate in hexane (v/v). FAHFAs were eluted with 5 ml of ethyl acetate, dried in a vacuum evaporator, and resuspended in 100 μl MeOH prior to measurement (43).

### Liquid chromatography-mass spectrometry (LC-MS)

The LC-MS system consisted of a Vanquish UHPLC system (Thermo Fisher Scientific) coupled to a Q Exactive Plus mass spectrometer (Thermo Fisher Scientific). Lipid profiling in positive and negative ion modes was performed as described before (22).

### Data analysis and statistics

LC-MS data were processed with the software MS-DIAL v5.5/MS-FINDER containing a predefined FAHFA library. Values are normalized per serum equivalent per fraction based on sequential sample dilution during ultracentrifugation protocol.

## Results

### BSA binding does not support albumin as the dominant FAHFA carrier

**Fig. 1:**
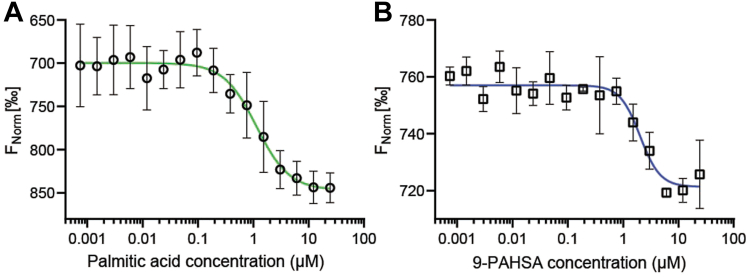
MST measurements of the interaction between BSA and PA (A) or 9-PAHSA (B). Unlabeled BSA (1 μM) was mixed with serially diluted PA or 9-PAHSA and measured. The error bars show the SEM from three technical replicates - representative of 3 different assays.

Serum albumin is a highly abundant protein and the main carrier of unesterified FA in blood, with its seven hydrophobic binding sites capable of binding FA. Albumin was the first tested potential FAHFA transporter in the blood. We utilized MST, a technique commonly used for protein-ligand interactions, but which has not yet been widely utilized for protein-lipid interactions (44, 45). First, the method was validated by measuring the interaction between BSA and PA (Fig. 1A) – an interaction which has been previously investigated using various techniques (46, 47, 48, 49). The measured dissociation constant (K_d_ = 1.140 ± 0.590 μM) was higher than previously reported (49).

As MST was found to be a suitable method for measuring the interaction between BSA and PA, it was used to investigate whether the transporter protein could also bind FAHFA (Fig. 1B). The dissociation constant measured for BSA and 9-PAHSA was found to be 2.025 ± 0.738 μM, indicating a weaker interaction than that observed for BSA-PA. Given that the concentration of 9-PAHSA in serum is in the nanomolar range in multiple species (50), and the concentration of palmitate is in the millimolar range, the BSA-9-PAHSA K_d_ values suggest that this interaction is not physiologically relevant. Therefore, the FAHFA is likely not abundant enough to bind to serum albumin in competition with other FAs but can be used in in vitro studies as a FAHFA carrier.

### FAHFAs are transported in the blood within lipoprotein particles

We next examined whether FAHFAs are transported by the TG-rich lipoproteins, chylomicrons, and VLDL. We isolated the particles using sequential ultracentrifugation, then performed lipidomics and proteomics by mass spectrometry to confirm particle identity and to assess FAHFA content.

**Fig. 2:**
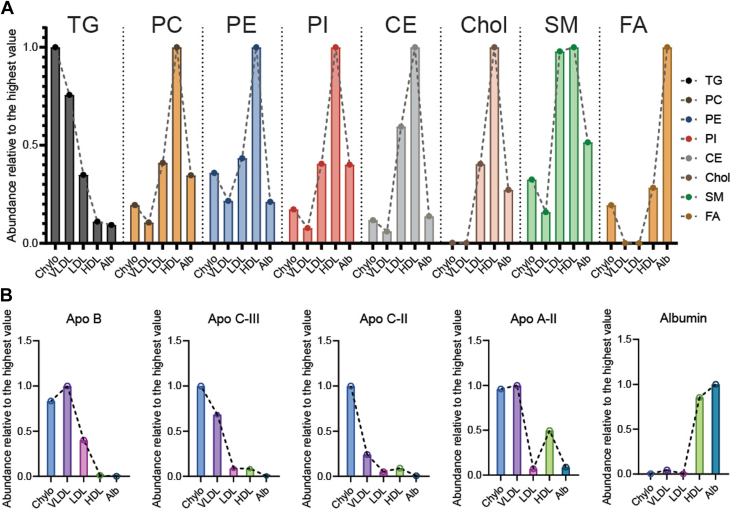
Lipidomic and proteomic characterization of serum lipoprotein fractions profile. The particles were purified by serial ultracentrifugation from the serum of mice fed HFD and gavaged with cream. A: Extracted lipids were analyzed by LC-MS. Specific lipid classes are color-coded per fraction. TG, triacylglycerols; PC, phosphatidylcholines; PE, phosphatidylethanolamines; PI, phosphatidylinositols; CE, cholesteryl esters; Chol, cholesterol; SM, sphingomyelins; FA, fatty acids. Bars represent normalized intensities of marker species. Marker lipids: TG 18:1/18:1/18:1; PC 16:0_18:1; PE 18:0_18:2; PI 16:0_18:1; CE 18:1; ST 27:1;O [cholesterol]; SM 18:1;O2/16:0; FA 16:0. Data are individual lipid species values per profile/fraction (n = 1), representative of 3 independent preparations. B: LC-MS characterization of the proteome. Bars represent peptide abundances normalized to the highest total peptide intensity. Data are individual values per profile/fraction [Chylomicrons > VLDL > LDL > HDL > Albumin] (n = 1), representative of 2 independent preparations. Proteomics does not distinguish between Apo B isoforms.

Lipidomics (Fig. 2) showed that lipoprotein particle purification was successful: PC, PE, PI, CE, and cholesterol were highly abundant in HDL as compared to chylomicrons, VLDL, or LDL. Chylomicrons and VLDL particles were rich in TG, as expected. The albumin fraction contained the majority of unesterified FAs, which is in line with its function as the major carrier of free FAs in blood.

Proteomics (Fig. 2B) further confirmed lipoprotein identities. Apo B-100 and Apo B-48 protein (Apo B in the figure) were found primarily in VLDL, LDL, and chylomicrons. Apo C-III and Apo C-II were found in the chylomicron, VLDL, LDL, and HDL fractions, while Apo A-II was found mostly in chylomicrons, VLDL, and HDL. Albumin was detected in the lipoprotein-depleted "albumin" fraction, but it was also found in the HDL fraction due to high serum albumin content. Overall, the combined lipidomics and proteomics data confirmed the identity of the sequentially purified lipoprotein particles.

### FAHFA and TG-EST are found primarily in chylomicrons, VLDL, and LDL

**Fig. 3:**
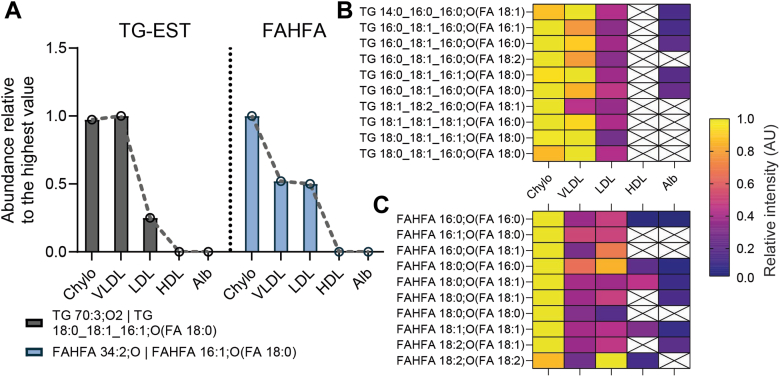
TG-EST and FAHFAs in lipoprotein particle fractions profile. The particles were purified by serial ultracentrifugation from the serum of mice fed HFD and gavaged with cream. A: Illustrative lipoprotein profiles for TG-EST containing FAHFA 34:2;O [FAHFA 16:1;O(FA 18:0)], and the FAHFA 34:2;O; n = 1 per fraction, representative of 3 profiles. B: Heat map of TG-EST levels in different fractions – horizontal axis – from Chylomicron to Albumin fraction. C: Heatmap of FAHFA presence in different fractions. Alb, albumin; Chylo, chylomicrons.

Lipidomics revealed the presence of TG-EST and FAHFAs in the lipoprotein particles of HFD-fed mice, which were gavaged with cream. Standard lipidomics profiling revealed the presence of FAHFA 16:1;O(FA 18:0), as well as TG-EST containing the same FAHFA in chylomicrons, VLDL, and LDL (Fig. 3A). While FAHFA 34:2;O [FAHFA 16:1;O(FA 18:0)] was the only FAHFA family detected using a general profiling approach, the data revealed the presence of various TG-EST mainly in chylomicrons, VLDL, and LDL (Fig. 3B). These results can be directly compared, as equivalent volume relative to the volume of the purified fraction of the lipoprotein sample was extracted. As only one FAHFA molecule was detected at low intensity by standard lipidomics profiling, the SPE-based FAHFA enrichment method was employed in an attempt to improve coverage by concentrating FAHFAs present within the lipoprotein particles. The results (Fig. 3C) show that a variety of FAHFA molecules were found within the chylomicron, VLDL, and LDL fractions, albeit at reduced intensity compared to TG-EST results from standard lipidomics.

### FAHFA and TG-EST are found in human chylomicrons

**Fig. 4:**
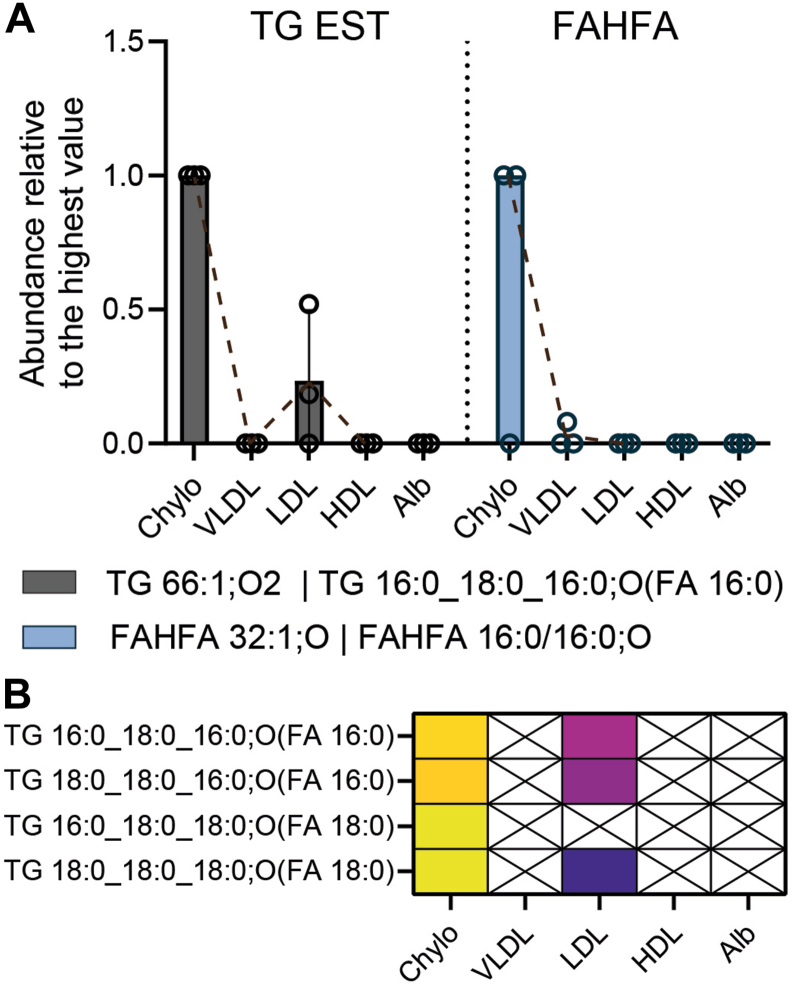
TG-EST and FAHFAs in lipoprotein particle fractions profile. The particles were purified by serial ultracentrifugation from the serum of human volunteers fed with cream. A: Illustrative lipoprotein profiles for TG-EST containing FAHFA 32:1;O [FAHFA 16:0;O(FA 16:0)], and the FAHFA 32:1;O; n = 3 per fraction. B: Heat map of TG-EST levels in different fractions—horizontal axis—from Chylomicron to Albumin fraction. Heatmap intensities share the same scale as Figure 3.

Next, three human volunteers drank a lipid bolus (33% cream) and blood collected 2 h after the ingestion was used for lipoprotein preparation following the murine protocol. Heavy whipping cream contains mainly saturated fatty acids, and so highly saturated TG-EST species were found in chylomicrons (Fig. 4). Only one saturated FAHFA 16:0/16:0;O was detected. This case study documents that dietary lipids, forming chylomicrons, transport FAHFA-containing lipid species also in humans.

### LPL hydrolyzes TG-EST

**Fig. 5:**
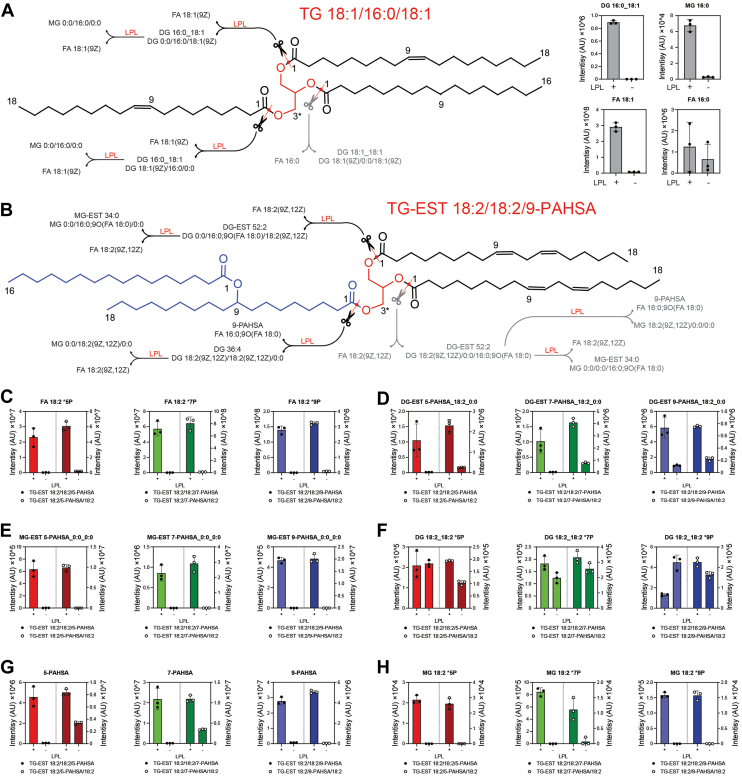
In vitro TG-EST hydrolysis by LPL. Lipoprotein-like particles containing TG-EST (with 5-, 7- or 9-PAHSA at *sn-2* or *sn*-1/-3 position) or TG 18:1/16:0/18:1 and Apo C-II were incubated with/without LPL, followed by lipidomics analysis of lipolysis products. A: Graphical illustration of theoretical TG lipolysis by LPL and its products. The grey scheme is the non-preferred theoretical pathway. LPL (+) marks native LPL, LPL (−) marks heat-inactivated LPL. B: Graphical illustration of theoretical TG-EST 18:2/18:2/9-PAHSA lipolysis by LPL and its products. The grey scheme is the non-preferred theoretical pathway. C–H: Levels of lipolysis products of LPL lipolysis of different TG-EST. Data are presented as means ± SEM (n = 3). ∗5P, ∗7P, and ∗9P indicate that the original substrate contained 5-, 7-, or 9-PAHSA, for clarity. Note the differences in Y axes ranges of the panels when comparing lipid levels.

LPL liberates the FAs from the TGs in circulating chylomicrons and VLDL (51, 52). Thus, it was important to establish whether LPL can hydrolyze the TG-EST present in these lipoproteins. An in vitro hydrolysis assay was performed, where lipoprotein-like artificial particles containing TG-EST with 5-, 7-, and 9-PAHSA or TG substrate were incubated with/without LPL, followed by lipidomic analysis. Figure 5A shows an example of the action of native LPL and heat-inactivated LPL on a TG substrate. LPL should hydrolyze the *sn*-1 and *sn*-3 positions of the molecule (TG or DG) and skip the *sn-*2 position. Symmetric TG substrate TG 18:1(9Z)/16:0/18:1(9Z) confirmed enzymatic activity and specificity, releasing mainly oleic acid and only traces of palmitic acid, leaving the DG 16:0_18:1 and MG 16:0 intermediates, while no DG 18:1_18:1 or MG 18:1 were detected.

Similarly, Figure 5B shows an example of the action of LPL on one of the TG-EST molecules used in this assay—LPL would preferably hydrolyze a TG-EST at the *sn*-1 and *sn*-3 positions of the molecule, based on previously-established *sn*-position preferences of the enzyme (53). LPL hydrolyzed all three TG-EST molecules used in the assay - the lipase readily liberated LA from all TG-EST molecules, thus the presence of FAHFA did not alter LPL enzymatic function (Fig. 5C). A high abundance of diacylglycerol estolide (DG-EST) containing each of the three FAHFA shows that LPL can hydrolyze a normal FA present within a TG-EST molecule (Fig. 5D). In line with the well-known LPL preference for FA at *sn*-1 or *sn-3* positions, there was more FAHFA-containing monoacylglycerol estolide (MG-EST) after hydrolysis of TG-EST containing the FAHFA at the *sn*-2 position as compared to TG-EST with FAHFA at the *sn*-1/-3 position (Fig. 5E, note the Y-axes ranges). However, LPL is also capable of hydrolyzing LA at *the sn*-2 position, leaving MG-EST with the FAHFA at the *sn*-1/-3 position as a product, albeit at a lower abundance. TG-EST can degrade in vitro, as indicated by DG 18:2_18:2's presence in samples with or without LPL (Fig. 5F). However, the overall intensity of this DG is relatively low. All other TG-EST reaction intermediates and products show vast differences between LPL+ and LPL- conditions, suggesting that TG-EST degradation does not have a significant effect on the results. Figure 5G further establishes LPL's ability to liberate FAHFAs from TG-EST and reveals that the lipase has different preferences for regioisomers. Interestingly, the distance between the glycerol ester bond and the FAHFA ester bond appears to influence the efficiency of the enzyme: the longer the distance, the higher the hydrolytic activity, suggesting a LPL sensitivity to steric hindrance at the glycerol ester bond. The enzyme appears to prefer cleaving 9-PAHSA at the *sn*-1/-3 position, with lower efficiency for *sn*-2 9-PAHSA. It shows a weaker preference for 7-PAHSA, with similar affinity for either *sn* position. LPL has the lowest preference for liberating 5-PAHSA, and prefers the *sn*-2 compared to the *sn*-1/-3 position. The MG 18:2 product was present under all conditions at low abundance compared to other products (Fig. 5H), implying that LPL can indeed hydrolyze FAHFA at *the sn*-2 or *sn*-1/-3 position of the TG-EST. Overall, the results identify LPL ability to hydrolyze TG-EST to liberate FAHFAs, with different regioisomer preferences. LPL hydrolysis of the FAHFA ester linkage was not investigated.

## Discussion

The FAHFAs have an ever-expanding list of health benefits, including anti-diabetic and anti-inflammatory effects (50, 54, 55). However, how the FAHFAs exert these effects physiologically remains unclear in part due to our lack of understanding of the factors that control their transport and delivery to particular cells or tissues. This knowledge gap was addressed as we reveal in this study that FAHFAs and the FAHFA-containing TG-EST are present in chylomicrons, VLDL, and LDL. Furthermore, we demonstrate that LPL can liberate the FAHFAs from lipoprotein-like particles in vitro, suggesting that regulation of LPL activity would influence FAHFA availability and tissue distribution.

Our data suggest that TG-ESTs, which store FAHFAs, are transported in chylomicrons and VLDL and delivered to tissues such as adipose tissue, a major site of FAHFA synthesis and storage (15, 22, 23). Dietary FAHFAs appear to be absorbed by enterocytes in the small intestine and incorporated into TG-ESTs before being packaged into chylomicrons. Because lymphatic lipids enter the circulation through the subclavian vein before passing through the liver, they initially bypass hepatic metabolism, which may increase their availability to peripheral tissues (31).

ATGL, an enzyme capable of generating TG-ESTs, is present in enterocytes (23, 56). However, current evidence suggests that it does not act on the pool of dietary triglycerides destined for chylomicron secretion (57, 58). Instead, ATGL is thought to hydrolyze a cytoplasmic triglyceride pool derived from the circulation, which provides local energy for enterocytes (58). These findings indicate that the mechanisms underlying dietary FAHFA absorption and processing in enterocytes, as well as the possible role of ATGL in TG-EST synthesis in the small intestine, require further investigation.

FAHFAs are also synthesized in the liver by ATGL (1, 15) and ATGL may also use its transacylase activity to incorporate FAHFAs into TG-ESTs, thereby enabling their transport in VLDL particles (23).

Our finding that LPL hydrolyzes TG-ESTs further suggests that regulation of LPL may be an important determinant of FAHFA delivery to specific tissues, since LPL is subject to complex, tissue-specific metabolic control (59). It remains unclear whether TG-EST hydrolysis by LPL is governed by the same regulatory factors that control the hydrolysis of other lipoprotein triglycerides.

## Conclusion

In summary, lipogenic organs like adipose tissue, liver and intestine can synthesize various FAHFA-containing lipids, but if and how they all contribute to the circulating FAHFA pool is still unknown. Here we show that FAHFA and TG-EST are associated with chylomicrons and VLDLs in the context of postprandial lipid metabolism and that LPL can liberate FAHFA from TG-EST.

### Limitations of the study

The detection of FAHFAs using lipidomic profiling is constrained by the lipoprotein particle separation, specifically the serial dilution with a concentrated NaBr solution. Consequently, only the major FAHFA peaks were reported, without resolution of individual regioisomers. High serum lipid load via HFD feeding and cream gavage/intake represents the postprandial state of lipid trafficking, dominated by chylomicrons and VLDL. Specific feeding regime (high fat diet > fasting > lipid bolus) was applied to maximize exogenous supply of lipids to enrich lipoprotein particles in FAHFA-containing lipids and to overcome analytical challenges. Only traces of FAHFA were detected in lipoprotein particles in mice fed a chow diet or after fasting these mice for 6 h due to sample dilution during lipoprotein separation. HFD feeding was necessary to overcome this technical challenge. Lymph sampling would be the optimal approach to analyze nascent chylomicrons. Advanced techniques for protein–lipid interaction should be applied to fully test dissociation constants of FAHFA isomers. Extracellular vesicles isolated from the whole serum were tested negative for FAHFA-containing lipids. However, tissue- or cell-specific extracellular vesicles could transport a FAHFA cargo, and this option has not been explored (SI Material).

## Associated content

Lipidomics Standards Initiative reporting checklist.

## Data availability

Proteomics and lipidomics data are available from ProteomeXchange with identifier PXD078529 and from the corresponding author upon reasonable request.

## Supplemental data

This article contains supplemental data (60, 61).

## Conflict of interest

The authors declare that they have no conflicts of interest with the contents of this article.

## Supplemental data

Supplemental Materials

LSI_checklist
