# Supplementary material for: FAHFAs are detected in postprandial chylomicron and VLDL fractions and can be released from TG estolides by LPL in vitro
Source: J Lipid Res. 2026 May 20;67(6):101059. doi: 10.1016/j.jlr.2026.101059 (PMC13285368; doi:10.1016/j.jlr.2026.101059)
Supplement: Supplemental Materials [file mmc1.docx]

**SUPPLEMENTAL INFORMATION**

**FAHFAs are detected in postprandial chylomicron and VLDL fractions and can be released from TG estolides by lipoprotein lipase in vitro**

Dovile Milonaityte^1^, Kristyna Brejchova^1^, Jaroslav Srp^2^, Eva Kudova^2^, Hana Chodounska^2^, Laurence Balas^3^, Thierry Durand^3^, Nada A Abumrad^4^, Ondrej Kuda^1^*

^1^Institute of Physiology, Czech Academy of Sciences, Videnska 1083, 14200 Prague, Czech Republic

^2^Institute of Organic Chemistry and Biochemistry, Czech Academy of Sciences, Flemingovo nam. 2, Prague 6, 166 10, Czech Republic

^3^Institut des Biomolécules Max Mousseron, UMR 5247, CNRS, Université Montpellier, ENSCM, Faculté de Pharmacie, Montpellier, France

^4^Department of Medicine, Division of Nutritional Sciences and Obesity Research, Washington University School of Medicine, St Louis, MO, USA

## Lipidomics profiles of serum extracellular vesicles

Extracellular vesicles (EVs) are particles released by cells into interstitial spaces and blood, and function in cell-cell communication and in the transfer of cargo, including lipids. The EVs comprise a lipid bilayer and an internal hydrophilic region (1). We examined whether EVs are capable of transporting FAHFA or TG-EST. Gradient ultracentrifugation was used to isolate mouse EVs, followed by lipidomics analysis. The lipid content of the collected fractions (Figure S1) showed the presence of TG, phospholipids (PC, PE, PI), cholesterol, CE, and sphingomyelin (SM). No FAHFAs or TG-ESTs were detected in any of the fractions.


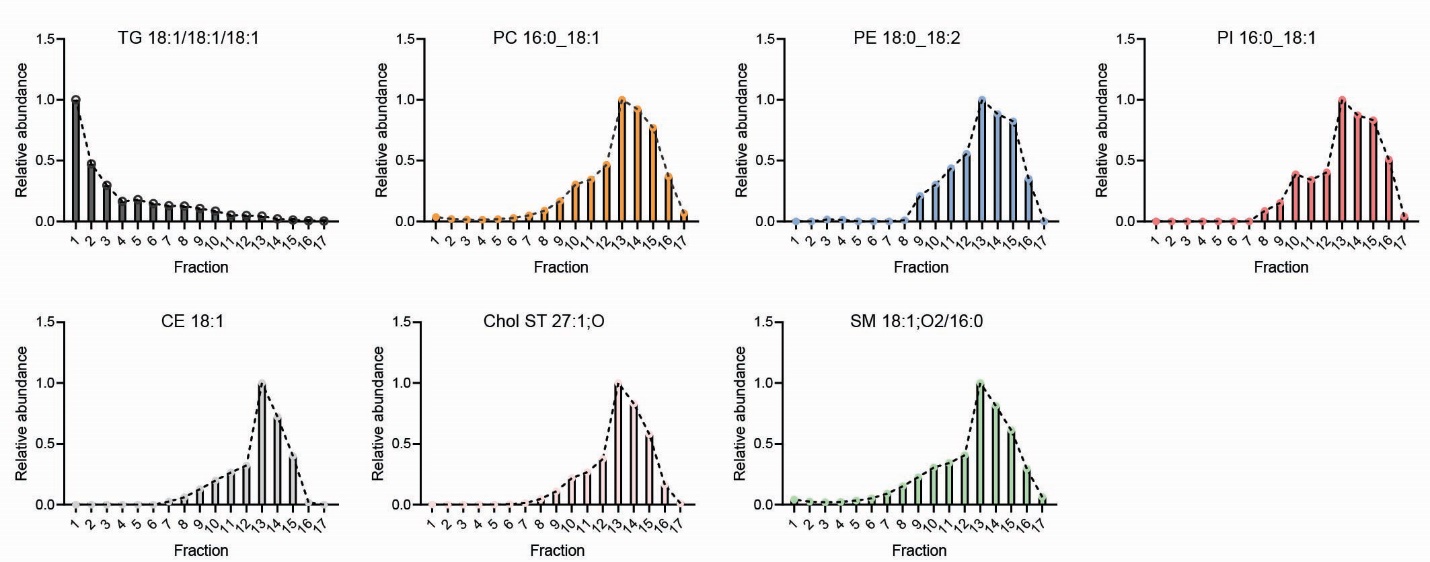


**Figure S1**: Lipidomics profile of EVs purified from mouse serum using gradient ultracentrifugation. Extracted lipids from each fraction were measured by LC-MS. TG, triacylglycerols; PC, phosphatidylcholine; PE, phosphatidylethanolamine; PI, phosphatidylinositol; CE, cholesteryl ester; Chol, cholesterol; SM, sphingomyelins. Marker lipids: TG 18:1/18:1/18:1; PC 16:0_18:1; PE 18:0_18:2; PI 16:0_18:1; CE 18:1; ST 27:1;O [cholesterol]; SM 18:1;O2/16:0. Data are values of individual lipid species per fraction, *n* = 1.

## Extracellular vesicle isolation

Previously published density gradient ultracentrifugation protocol (2) was used to purify serum extracellular vesicles (EVs) with some modifications. Pooled mouse serum was centrifuged at 2,000 × *g* for 10 min, followed by a centrifugation at 16 000 × *g* for 30 min to remove cellular debris. The debris was further removed by passing the serum through a 0.22 µm syringe filter. Pre-clarified mouse serum (500 µL) was mixed with 900 µL of homogenization buffer (1.5 M sucrose, 6 mM EDTA, 60 mM Tris-HCl). Iodixanol solution (60%; 2.833 mL) (Sigma-Aldrich, D1556) was added to the serum mixture to produce a 40% iodixanol solution containing serum. The solution was transferred to the bottom of a thin-wall polycarbonate tube (Beckman Coulter, 344061). A discontinuous gradient was created by overlaying 4.25 ml of the 20% iodixanol, followed by an overlay of 4.25 ml of the 10% iodixanol, and a final overlay of 4.25 ml of the 5% iodixanol solutions produced by mixing the 60% iodixanol with an appropriate volume of homogenization buffer. Tubes were spun in a Beckman Coulter Optima XPN-90 ultracentrifuge with a SW 32.1 Ti rotor at 100 000 × *g* for 19 hours (7 Acceleration, 5 Deceleration). Fractions were collected (1 ml each) and used for further experiments.

## References

1. Gurung, S., D. Perocheau, L. Touramanidou, and J. Baruteau. 2021. The exosome journey: from biogenesis to uptake and intracellular signalling. *Cell Communication and Signaling* **19**: 47.

2. Li, K., D. K. Wong, K. Y. Hong, and R. L. Raffai. 2018. Cushioned-Density Gradient Ultracentrifugation (C-DGUC): A Refined and High Performance Method for the Isolation, Characterization, and Use of Exosomes. *Methods in Molecular Biology* **1740**: 69-83.
