# Supplementary material for: FAHFAs are detected in postprandial chylomicron and VLDL fractions and can be released from TG estolides by LPL in vitro
Source: J Lipid Res. 2026 May 20;67(6):101059. doi: 10.1016/j.jlr.2026.101059 (PMC13285368; doi:10.1016/j.jlr.2026.101059)
Supplement: LSI_checklist [file mmc2.pdf]

Contents of Report

Created by https://lipidomicstandards.org, version v2.5.0

Separation Workflow 1
- Overall study design 1
- Lipid extraction 1
- Analytical platform 1
- Quality control 2
- Method qualification and validation 2
- Reporting 2

Sample Descriptions 2
- Serum lipoprotein fractions / Mouse / Serum 2

Lipid Class Descriptions 2
- 1) FA[M-H]- / Lipid identification 2
- 1) FA[M-H]- / Lipid quantification 3
- 2) PC[M-H]- / Lipid identification 3
- 2) PC[M-H]- / Lipid quantification 3
- 3) PE[M-H]- / Lipid identification 4
- 3) PE[M-H]- / Lipid quantification 4
- 4) PI[M-H]- / Lipid identification 4
- 4) PI[M-H]- / Lipid quantification 4
- 5) CE[M+NH4]+ / Lipid identification 5
- 5) CE[M+NH4]+ / Lipid quantification 5
- 6) ST / Lipid identification 5
- 6) ST / Lipid quantification 6
- 7) SM[M-H]- / Lipid identification 6
- 7) SM[M-H]- / Lipid quantification 6
- 8) FAHFA[M-H]- / Lipid identification 6
- 8) FAHFA[M-H]- / Lipid quantification 7
- 9) TG-EST[M+NH4]+ / Lipid identification 7
- 9) TG-EST[M+NH4]+ / Lipid quantification 7
- 10) TG[M+NH4]+ / Lipid identification 7
- 10) TG[M+NH4]+ / Lipid quantification 8

Separation Workflow

Overall study design

Title of the study	Lipidomics profiling		
Document creation date	10/11/2025	Principal investigator	Ondrej Kuda
Institution	Institute of Physiology, CAS	Corresponding Email	ondrej.kuda@fgu.cas.cz
Is the workflow targeted or untargeted?	Untargeted	Clinical	No

Lipid extraction

Extraction method	2-phase system	pH adjustment	None
2-phase system	MTBE	Special conditions	on ice
Were internal standards used?	Yes	Deposition method	protocol
Internal standards used	[13C4]-9-PAHSA, CUDA, and IS in doi: 10.1007/s00018-025-05783-w		

Analytical platform

Ionizaton additives	Ammonium formate, Formic acid	Number of separation dimensions	One dimension
Separation type 1	LC	Separation mode 1 (liquid)	RP
Detector	Mass spectrometer	MS type	Orbitrap

MS vendor	Thermo	Ion source	ESI
MS Level	MS^1, MS^2	Mass resolution for detected ion at MS^1	High resolution
Resolution at m/z 200 at MS^1	140000	Mass accuracy in ppm at MS^1	2
Recording mode of raw data at MS^1	Centroid mode	Mass window for precursor ion isolation (in Da total isolation window)	1
Mass resolution for detected ion at MS^2	High resolution	Resolution at m/z 200 at MS^2	17500
Mass accuracy in ppm at MS^2	5	Recording mode of raw data at MS^2	Centroid mode
Was/Were additional dimension/techniques used	No		

Quality control

Blanks	Yes	Type of Blanks	Extraction blank, Solvent blank
Quality control	No		

Method qualification and validation

Method validation	No

Reporting

Are reported raw data uploaded into repository?	Available on request	Are metadata available?	Yes
Raw data upload	Available on request		

Sample Descriptions

Serum lipoprotein fractions / Mouse / Serum

Storage and collection conditions	Available	Provided preanalytical information	Time to separate plasma/serum (min)
Time to separate plasma/serum (min)	30	Temperature handling original sample	4-8 °C
Instant sample preparation	Yes	Storage temperature	4-8 °C
Additives	NaCl, NaBr density gradient	Were samples stored under inert gas?	No
Additional preservation methods	No	Biobank samples	No

Lipid Class Descriptions

1) FA[M-H]- / Lipid identification

Lipid class	FA	MS Level for identification	MS^1, MS^2
Identification level	Species level	MS^1 adduct	[M-H]-
Isotope correction at MS^1	Type 2	MS^2 adduct	[M-H]-

Fragments for identification

Fragment name
MLF

Isotope correction at MS^2	Type 2	MS^1 verified by standard	Yes
MS^2 verified by standard	Yes	Background check at MS^1	Yes
Background check at MS^2	No	Did you presume assumptions for identification?	No
Limit of detection	No	RT verified by standard	Yes
Separation of isobaric/isomeric interference confirmed	Yes	Model for separation prediction	Yes
Lipid Identification Software	MS-DIAL	Data manipulation	Smoothing, Background subtraction
Nomenclature for intact lipid molecule	Yes	Nomenclature for fragment ions	N/A

1) FA[M-H]- / Lipid quantification

Quantitative	No	Normalization to reference	Yes
Batch correction	No		

2) PC[M-H]- / Lipid identification

Lipid class	PC	MS Level for identification	MS^1, MS^2
Identification level	Molecular species level	MS^1 adduct	[M-H]-
Isotope correction at MS^1	Type 2	MS^2 adduct	[M-H]-

Fragments for identification

Fragment name
MLF

Isotope correction at MS^2	Type 2	MS^1 verified by standard	No
MS^2 verified by standard	Yes	Background check at MS^1	Yes
Background check at MS^2	Yes	Did you presume assumptions for identification?	No
Limit of detection	No	RT verified by standard	Yes
Separation of isobaric/isomeric interference confirmed	Yes	Model for separation prediction	Yes
Lipid Identification Software	MS-DIAL	Data manipulation	Smoothing, Background subtraction
Nomenclature for intact lipid molecule	Yes	Nomenclature for fragment ions	N/A

2) PC[M-H]- / Lipid quantification

Quantitative	No	Normalization to reference	Yes
Batch correction	No		

3) PE[M-H]- / Lipid identification

Lipid class	PE	MS Level for identification	MS^1, MS^2
Identification level	Molecular species level	MS^1 adduct	[M-H]-
Isotope correction at MS^1	Type 2	MS^2 adduct	[M-H]-

Fragments for identification

Fragment name
MLF

Isotope correction at MS^2	Type 2	MS^1 verified by standard	Yes
MS^2 verified by standard	Yes	Background check at MS^1	Yes
Background check at MS^2	Yes	Did you presume assumptions for identification?	No
Limit of detection	No	RT verified by standard	Yes
Separation of isobaric/isomeric interference confirmed	Yes	Model for separation prediction	Yes
Lipid Identification Software	MS-DIAL	Data manipulation	Smoothing, Background subtraction
Nomenclature for intact lipid molecule	Yes	Nomenclature for fragment ions	N/A

3) PE[M-H]- / Lipid quantification

Quantitative	No	Normalization to reference	Yes
Batch correction	No		

4) PI[M-H]- / Lipid identification

Lipid class	PI	MS Level for identification	MS^1, MS^2
Identification level	Molecular species level	MS^1 adduct	[M-H]-
Isotope correction at MS^1	Type 2	MS^2 adduct	[M-H]-

Fragments for identification

Fragment name
MLF

Isotope correction at MS^2	Type 2	MS^1 verified by standard	Yes
MS^2 verified by standard	Yes	Background check at MS^1	Yes
Background check at MS^2	Yes	Did you presume assumptions for identification?	No
Limit of detection	No	RT verified by standard	Yes
Separation of isobaric/isomeric interference confirmed	Yes	Model for separation prediction	Yes
Lipid Identification Software	MS-DIAL	Data manipulation	Smoothing, Background subtraction
Nomenclature for intact lipid molecule	Yes	Nomenclature for fragment ions	N/A

4) PI[M-H]- / Lipid quantification

Quantitative	No	Normalization to reference	Yes
Batch correction	No		

5) CE[M+NH4]+ / Lipid identification

Lipid class	CE	MS Level for identification	MS^1, MS^2
Identification level	Molecular species level	MS^1 adduct	[M+NH4]+
Isotope correction at MS^1	Type 2	MS^2 adduct	[M+NH4]+

Fragments for identification

Fragment name
MLF

Isotope correction at MS^2	Type 2	MS^1 verified by standard	Yes
MS^2 verified by standard	Yes	Background check at MS^1	Yes
Background check at MS^2	Yes	Did you presume assumptions for identification?	No
Limit of detection	No	RT verified by standard	Yes
Separation of isobaric/isomeric interferece confirmed	Yes	Model for separation prediction	Yes
Lipid Identification Software	MS-DIAL	Data manipulation	Smoothing, Background subtraction
Nomenclature for intact lipid molecule	Yes	Nomenclature for fragment ions	N/A

5) CE[M+NH4]+ / Lipid quantification

Quantitative	No	Normalization to reference	Yes
Batch correction	No		

6) ST / Lipid identification

Lipid class	ST	MS Level for identification	MS^1, MS^2
Identification level	Molecular species level	Isotope correction at MS^1	Type 2

Fragments for identification

Fragment name
MLF

Isotope correction at MS^2	Type 2	MS^1 verified by standard	Yes
MS^2 verified by standard	Yes	Background check at MS^1	Yes
Background check at MS^2	Yes	Did you presume assumptions for identification?	No
Limit of detection	No	RT verified by standard	Yes
Separation of isobaric/isomeric interferece confirmed	Yes	Model for separation prediction	Yes
Lipid Identification Software	MS-DIAL	Data manipulation	Smoothing, Background subtraction
Nomenclature for intact lipid molecule	Yes	Nomenclature for fragment ions	N/A

6) ST / Lipid quantification

Quantitative	No	Normalization to reference	Yes
Batch correction	No		

7) SM[M-H]- / Lipid identification

Lipid class	SM	MS Level for identification	MS^1, MS^2
Identification level	Molecular species level	MS^1 adduct	[M-H]-
Isotope correction at MS^1	No	MS^2 adduct	[M-H]-

Fragments for identification

Fragment name
MLF

Isotope correction at MS^2	Type 2	MS^1 verified by standard	Yes
MS^2 verified by standard	Yes	Background check at MS^1	Yes
Background check at MS^2	Yes	Did you presume assumptions for identification?	No
Limit of detection	No	RT verified by standard	Yes
Separation of isobaric/isomeric interferece confirmed	Yes	Model for separation prediction	Yes
Lipid Identification Software	MS-DIAL	Data manipulation	Smoothing, Background subtraction
Nomenclature for intact lipid molecule	Yes	Nomenclature for fragment ions	N/A

7) SM[M-H]- / Lipid quantification

Quantitative	No	Normalization to reference	Yes
Batch correction	No		

8) FAHFA[M-H]- / Lipid identification

Lipid class	FAHFA	MS Level for identification	MS^1, MS^2
Identification level	Molecular species level	MS^1 adduct	[M-H]-
Isotope correction at MS^1	Type 2	MS^2 adduct	[M-H]-

Fragments for identification

Fragment name
MLF

Isotope correction at MS^2	Type 2	MS^1 verified by standard	Yes
MS^2 verified by standard	Yes	Background check at MS^1	Yes
Background check at MS^2	Yes	Did you presume assumptions for identification?	No
Limit of detection	No	RT verified by standard	Yes
Separation of isobaric/isomeric interferece confirmed	Yes	Model for separation prediction	Yes
Lipid Identification Software	MS-DIAL	Data manipulation	Smoothing, Background subtraction

Nomenclature for intact lipid molecule	Yes	Nomenclature for fragment ions	N/A

8) FAHFA[M-H]- / Lipid quantification

Quantitative	No	Normalization to reference	Yes
Batch correction	No		

9) TG-EST[M+NH4]+ / Lipid identification

Lipid class	TG-EST	MS Level for identification	MS^1, MS^2
Identification level	Molecular species level	MS^1 adduct	[M+NH4]+
Isotope correction at MS^1	Type 2	MS^2 adduct	[M+NH4]+

Fragments for identification

Fragment name
MLF

Isotope correction at MS^2	Type 2	MS^1 verified by standard	Yes
MS^2 verified by standard	Yes	Background check at MS^1	Yes
Background check at MS^2	Yes	Did you presume assumptions for identification?	No
Limit of detection	No	RT verified by standard	Yes
Separation of isobaric/isomeric interferece confirmed	Yes	Model for separation prediction	Yes
Lipid Identification Software	MS-DIAL	Data manipulation	Smoothing, Background subtraction
Nomenclature for intact lipid molecule	Yes	Nomenclature for fragment ions	N/A

9) TG-EST[M+NH4]+ / Lipid quantification

Quantitative	No	Normalization to reference	Yes
Batch correction	No		

10) TG[M+NH4]+ / Lipid identification

Lipid class	TG	MS Level for identification	MS^1, MS^2
Identification level	Molecular species level	MS^1 adduct	[M+NH4]+
Isotope correction at MS^1	Type 2	MS^2 adduct	[M+NH4]+

Fragments for identification

Fragment name
MLF

Isotope correction at MS^2	Type 2	MS^1 verified by standard	Yes
MS^2 verified by standard	Yes	Background check at MS^1	Yes
Background check at MS^2	Yes	Did you presume assumptions for identification?	No

Limit of detection	No	RT verified by standard	Yes
Separation of isobaric/isomeric interferece confirmed	Yes	Model for separation prediction	Yes
Lipid Identification Software	MS-DIAL	Data manipulation	Smoothing, Background subtraction
Nomenclature for intact lipid molecule	Yes	Nomenclature for fragment ions	N/A

10) TG[M+NH4]+ / Lipid quantification

Quantitative	No	Normalization to reference	Yes
Batch correction	No		
